# A review on the effects of current chemotherapy drugs and natural agents in treating non–small cell lung cancer

**DOI:** 10.1051/bmdcn/2017070423

**Published:** 2017-11-24

**Authors:** Chih-Yang Huang, Da-Tong Ju, Chih-Fen Chang, P. Muralidhar Reddy, Bharath Kumar Velmurugan

**Affiliations:** 1 Graduate Institute of Basic Medical Science, China Medical University Taichung 404 Taiwan; 2 Graduate Institute of Chinese Medical Science, China Medical University Taichung 404 Taiwan; 3 Department of Biological Science and Technology, Asia University Taichung 413 Taiwan; 4 Department of Neurological Surgery, Tri-Service General Hospital, National Defense Medical Center Taipei 114 Taiwan; 5 Department of Internal Medicine, Division of Cardiology, Armed Forces Taichung General Hospital Taichung 406 Taiwan; 6 Department of Chemistry, Nizam College, Osmania University Hyderabad-500001 India; 7 Faculty of Applied Sciences, Ton Duc Thang University Tan Phong Ward, District 7 700000 Ho Chi Minh City Vietnam

**Keywords:** NSCLC, Chemotherapy, Solid tumors, Natural compounds, Surgery, Radiation

## Abstract

Lung cancer is the leading cause of cancer deaths worldwide, and this makes it an attractive disease to review and possibly improve therapeutic treatment options. Surgery, radiation, chemotherapy, targeted treatments, and immunotherapy separate or in combination are commonly used to treat lung cancer. However, these treatment types may cause different side effects, and chemotherapy-based regimens appear to have reached a therapeutic plateau. Hence, effective, better-tolerated treatments are needed to address and hopefully overcome this conundrum. Recent advances have enabled biologists to better investigate the potential use of natural compounds for the treatment or control of various cancerous diseases. For the past 30 years, natural compounds have been the pillar of chemotherapy. However, only a few compounds have been tested in cancerous patients and only partial evidence is available regarding their clinical effectiveness. Herein, we review the research on using current chemotherapy drugs and natural compounds (Wortmannin and Roscovitine, *Cordyceps militaris*, Resveratrol, OSU03013, Myricetin, Berberine, Antroquinonol) and the beneficial effects they have on various types of cancers including non-small cell lung cancer. Based on this literature review, we propose the use of these compounds along with chemotherapy drugs in patients with advanced and/or refractory solid tumours.

## 1. Introduction

### 1.1. Lung Cancer

Among all forms of cancer, lung cancer is the leading cause of mortality worldwide [1, 2]. It accounts for 1.4 million (or 17.7%) of all annual cancer deaths. Lung adenocarcinoma is the most common kind of lung cancer, found in both smokers and non-smokers, as well as those under the age of 45. Adenocarcinoma accounts for about 30 percent of primary lung tumours in male smokers and 40 percent in female smokers. Among non-smokers, these percentages approach 60 percent in males and 80 percent in females. This particular kind of lung cancer is also more common among Asian populations (www.cancer.gov). A rising death rate from lung cancer has been observed in Taiwan. Between 1971 and 2001, mortality rates per 100,000 per year of age-standardized lung cancer in Taiwan increased sharply, from 12.66 to 32.93 among men and from 7.83 to 14.94 among women. To date, in Taiwan, lung cancer is the leading cause of cancer deaths among women and the second leading cause among men [3].

Despite advances in development of new treatment modalities, the overall 5-year survival rate has only slightly increased over 2.5 decades, remaining at approximately 16% [4, 5]. Early diagnostic procedures and hits, and effective screening for non-small cell lung cancer (NSCLC) is still lacking [6]. Unfortunately, many patients with lung cancer are diagnosed at a late stage *(i.e.* stage III b or IV), and there is no curative treatment for such an advanced stage [7]. In Taiwan, liver, lung, stomach, colon, and oral cavity cancers are the five leading cancers responsible for cancer deaths among males; while lung, liver, cervix uteri, breast, and stomach are the five leading cancers responsible for cancer deaths among females [8]. Major lifestyle variables associated with an increased cancer risk in Taiwan include habits like cigarette smoking, alcohol drinking, and betel nut chewing. Cigarette smoking has been found to increase the risk of lung, Hepatoma Cellular Carcinoma, Oral cavity, Neural progenitor cells, Esophageal, Urinary bladder, and cervical cancer in a dose–response relationship [9-12]. Similarly, in western countries tobacco smoking is also one of the main etiological factor accounting for 85% of all lung cancer cases [13]. Studies carried out on the role of one’s diet as a potential risk factor for lung cancer have provided evidence that a higher dietary intake of fruits or vegetables is correlated with a lower risk of lung cancer. As only 10-15% of (heavy) smokers develop lung cancer, endogenous factors are thought to play an important role as well. Although lung cancer rarely results from inherited mutations of oncogenes or tumour suppressor genes, it has been related to a decreased capacity to detoxify certain types of cancer-causing chemicals or tobacco carcinogens. Notably, decreased DNA repair capacity and/or increasing cellular susceptibility to the accumulation of mutations, have been found to be independent risk factors specifically for the development of NSCLC [14].

There are two main types of lung cancer: non-small cell lung cancer (NSCLC) and small cell lung cancer (SCLC), and 80- 85 % of diagnoses are for non-small cell lung cancer [15]. The cancer cells of each type grow and spread *via* different ways. Based on histological types, NSCLC is primarily classified as squamous cell carcinoma, adenocarcinoma, or large-cell carcinoma [16]. Other less prevalentsubtypes, such as bronchioloalveolar carcinoma (BAC), comprise only 3-4% of cases, with 10-15% of adenocarcinomas having BAC features [17]. SCLC represents about 15-20% of lung cancer cases that present neuroendocrine morphological features [18].

### 1.2. Therapy for lung cancer

Treatment of any cancer aims to remove or destroy the cancerous cells without killing normal cells. The most common types of treatment for cancer include surgery, radiation, and chemotherapy which can be used either alone or in combination with each other or other therapies. Surgery involves removing the obvious cancerous tissue and it is the primary treatment for most cancers, particularly solid tumours. Ultrasonic and/or CD scanners are used as diagnostic tools to confirm a biochemical diagnosis and further determine the extent and spread of the tumour. Radiation therapy is the application of high energy X-rays to shrink a tumour. It is mostly used in conjunction with surgery or alternative chemotherapy or as a neo-adjuvant therapy to aid in surgery by reducing the size of a tumour and is considered local treatment since it affects only the tumour region. However, the therapeutic efficacy of radiotherapy alone for treating locally or regionally advanced cancer is often limited by tumour radio-resistance, systemic tumour progression, and local or distant metastases [19, 20]. Chemotherapy will be discussed in detail below in Section Two.

NSCLC is diagnosed at an advanced/ stage in a majority of patients for whom systemic therapy remains the basis of treatment [21, 22], even though treatment outcomes for NSCLC are still considered to be disappointing [23, 24]. Thus, NSCLC remains one of the most threatening cancers to treat. Recent advances have enabled scientists to better investigate the potential use of natural compounds for the treatment or control of various cancerous diseases. Prior to 1990, only a few cytotoxic drugs had confirmed activity against NSCLC, but a series of trials established platinum-based chemotherapy with taxanes (paclitaxel and doc-etaxel), vinca alkaloids (vinorelbine), and gemcitabine as the most effective treatment regimen [5]. But these chemotherapy-based regimens appear to have reached a therapeutic plateau; hence, effective, better-tolerated treatments are needed to overcome this issue [25].

## 2. Chemotherapy Drugs and treatment

Genomic studies have been promoting an effective application of anticancer drugs and many anticancer reagents in lung cancer chemotherapy treatment have progressively become ineffective for patients with a positive mutation of the tumour marker p53 [26]. Chemotherapy is the application of chemicals or drugs to kill cancer cells, and its effects are systemic. So far, there are several different classes of anticancer drugs based on their mechanisms of action, and they include the following: a) alkylating agents which damage DNA; b) anti-metabolites that replace the normal building blocks of RNA and DNA; c) antibiotics that interfere with the enzymes involved in DNA replication; d) topoisomerase inhibitors that inhibit either topoisomerase I or II, which are the enzymes involved in unwinding DNA during replication and transcription; e) mitotic inhibitors that inhibit mitosis and cell division; and f) corticosteroids, which are used for the treatment of cancer and to relieve the side effects from other drugs (Table 1).

**Table 1:** - Current regimen of treatment for lung cancer.

Drug name		Generic name		Use
Xeloda		Capecitabine		anti-metabolites
Avastin		Bevacizumab		VEGF/VEGFR inhibitors
Tarceva		Erlotinib		EGFR inhibitors
Cytoxan		Cyclophosphamide		alkylating agents
Taxol		Paclitaxel		mitotic inhibitorsa
Taxotere		Docetaxel		mitotic inhibitors
Gemzar		Gemcitabine		antimetabolites
Erbitux		Cetuximab		EGFR inhibitors
Alimta		Pemetrexed		antimetabolites
Navelbine		Vinorelbine		mitotic inhibitors
Platinol		Cisplatin		alkylating agents
Trexall		Methotrexate		antimetabolites, antipsoriatics, antirheumatics
Ethyol		Amifostine		antineoplastic detoxifying agents
Iressa		Gefitinib		EGFR inhibitor
Neosar		Cyclophosphamide		alkylating agents
Platinol-AQ		Cisplatin		alkylating agents
Photofrin		Porfimer		miscellaneous antineoplastics
Onxol		Paclitaxel		mitotic inhibitors

(http://www.drugs.com)

Patients with unresectable and metastatic cancer may benefit from (palliative) chemotherapy. According to current guidelines, first-line chemotherapeutic treatment consists of a platinum agent-based doublet, *e.g.* cisplatin or carboplatin in combination with a third-generation cytotoxic drug, gemcitabine, a taxane (paclitaxel, docetaxel), or vinorelbine. Meta-analyses of randomized clinical trials comparing cisplatin with carboplatin suggest that the clinical outcome of cisplatin doublets is slightly superior to carboplatin-based chemotherapy without being associated with an increase in severe toxic effects [27, 28]. Another meta-analysis showed a reduction in overall mortality in gemcitabine-platinum regimens as compared to platinum-based comparator regimens [29]. In late 2006, bevacizumab, a monoclonal antibody directed against vascular endothelial growth factor (VEGF), was approved in combination with paclitaxel and carboplatin chemotherapy for first-line treatment of patients with non-squamous NSCLC [30] [31]. Several anticancer drugs applied to the treatment of lung cancer (bleomycin, doxorobucin, etoposide (VP-16), cisplatin, and methotrexate) have been reported to enhance Fas ligand (FasL) expression on the surface of Fas receptor–expressing cells, suggesting that apoptosis caused by these drugs may be mediated by means of Fas cross-linking [32, 33]. Platinum drugs are effective for patients with a positive K-ras mutation, while a number of drugs are not useful for those with increased Her-2 expression. In addition, an increased expression of p27 enhances the efficacy of taxanes [34], while taxanes are ineffective for patients with a positive mutation of beta-tubulin. In conclusion, cisplatin and other platinum drugs would not benefit patients who have a high excision repair protein (ERCC1) expression [35].

### 2.1. Effects of Cisplatin

Cisplatin (cis-diamminedichloroplatinum, DDP) is among the most effective and widely used chemotherapeutic agents employed for treatment of solid tumours. It is a platinum-based compound that forms intra- and inter-strand adducts with DNA, and thus it is a potent inducer of cell cycle arrest and apoptosis in most cancer cell types [36]. Unfortunately, many patients with these malignancies eventually relapse and become refractory (drug resistant) to chemotherapy either intrinsically (*e.g.* as observed in patients with colorectal, lung, and prostate cancer) or acquired following cisplatin chemotherapy (as often seen in patients with ovarian cancer) [37, 38]. Cancer cells can develop cisplatin resistance through changes in (1) drug transport leading to reduced intracellular cisplatin accumulation, (2) an enhanced drug detoxification system due to elevated levels of intracellular scavengers such as glutathione and/or metallothioneins, (3) changes in DNA repair involving increased nucleotide excision repair, inter-strand crosslink repair or loss of mismatch repair, (4) changes in DNA damage tolerance mechanisms, and finally (5) changes in the apoptotic cell death pathways [39-42]. Cisplatin resistance might result in conjunction with GSH followed by the inactivation of cisplatin or the prevention of cisplatin-adducts formation. The level of GST-π isoenzyme expression has been found to be significantly associated with intrinsic resistance to cisplatin in lung cancer cell lines [43].

### 2.2. Effects of Taxanes

The chemotherapeutic agents known as taxanes have emerged as one of the most powerful classes of compounds to combat cancer, exhibiting a wide range of activity. The tubulin/microtubule complex has been proven to be a clinically useful antitumor target. The examples of chemotherapeutics that act *via* perturbation of tubulin polymerization include paclitaxel (Taxol®), docetaxel (Taxotere®), vinblastine, and discodermolide. First, docetaxel is a semi-synthetic derivative of paclitaxel. Next, vinblastine, unlike the other three compounds that all stabilize microtubules, aggregates tubulin and leads to microtubule depolymerisation [43-46].

**Fig. 1:**
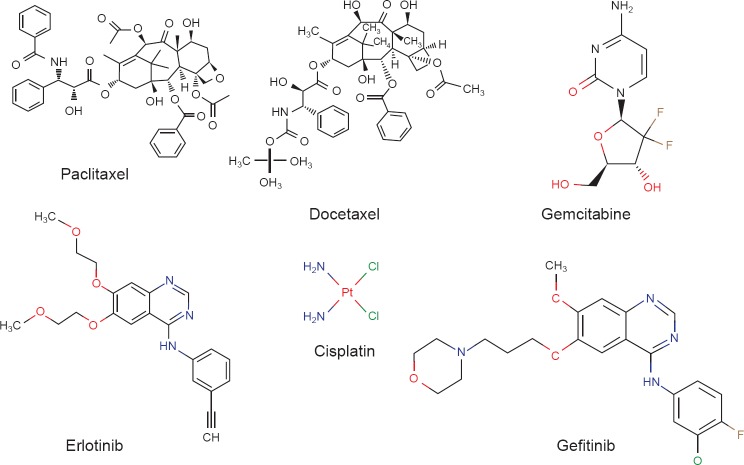
Chemical structures of anti-tumour agents with clinical applications.

Randomized clinical trials evaluating docetaxel and paclitaxel in a first-line treatment setting for metastatic breast, lung, ovarian, and digestive cancers, as well as in the adjuvant setting for breast cancer, have confirmed that taxanes are leading contributors to the armamentarium of cancer treatments [47]. Though the taxanes share similar mechanisms of action, differences are apparent in their molecular pharmacology, pharmacokinetics, and pharmacodynamic profiles. These differences may account for the differences observed between the taxanes in their clinical activity and toxicity (Fig. 1).

#### Paclitaxel

Paclitaxel is used as a first-line chemotherapy treatment for NSCLC, but patients' acquired resistance becomes a critical problem. Tubulin is the “building block” of microtubules, and agents that bind to tubulin are believed to block cell division by interfering with the function of the mitotic spindle, blocking the cells at the metaphase-anaphase junction of mitosis. Microtubules are complex structures involved in numerous cellular functions, including the maintenance of cell shape, intracellular transport, secretion, and neurotransmission. Moreover, microtubules are highly dynamic and unstable structures that are constantly incorporating free dimers and releasing dimers into the soluble tubulin pool [47]. Chang *et al.* (1993) and Murphy *et al.* (1993) have evaluated a 24-h infusion schedule (regimen) of paclitaxel in the treatment of advanced and metastatic NSCLC and yielded response rates of 21% and 24% respectively [48, 49], while short infusion schedules for 3 h and 1 h yielded similar results [50, 51]. Docetaxel exhibits greater affinity to ß-tubulin, targeting centrosome organization and acting on cells during three phases of the cell cycle (S/G2/M), whereas paclitaxel causes cell dam age by affecting the mitotic spindle in the G2 and M phases of the cell cycle, and maximum resistance to paclitaxel is early in the S phase [52, 53]. Recently a report has shown that concentrations of paclitaxel >10 nM inhibit endothelial cell proliferation through a G2-M arrest and induce subsequent cell death by apoptosis, similar to the effects it has on tumour cell lines [54]. During the last decade, additional activities of taxol have been described in many aspects including taxol-induced phosphorylation of I-κBα, while several studies have also shown that taxol can directly activate survival pathways such as Bcl-2, Akt, Cox-2, mitogenactivated protein kinase, *etc.,* independent of NF-κB [55, 56].

The phosphorylation of Bcl-2 appears to be a hallmark of cell death induced by paclitaxel, but the correlation between this event, mitotic arrest, and apoptosis remains controversial. Initial reports have suggested that the phosphorylation of Bcl-2 leads to inactivation of its antiapoptotic function [57]. Currently, the molecular mechanism by which paclitaxel-induced mitotic arrest leads to apoptosis is not clear, although evidence for the involvement of several signalling pathways has been shown including the action of various protein kinases such as mitogen-activated protein kinases (MAPK), serine/threonine kinase-dependent phosphorylation of Bcl-2, and the p53 pathway [58]. Activation of the caspase-9/Apaf-1/cytochrome c apoptosome in the mitochondrialdependent apoptotic pathway leads in turn to caspase-3 activation [59]. Ofir *et al.* (2002) have also found that procaspase-9 is not activated after taxol treatment in MCF-7 breast cancer and SKOV3 ovarian cancer cell lines, and this drug induces apoptosis independently of caspases-3 and -9 [60]. However, during taxol-induced apoptosis in CCRF-HSB-2 cells (Human Caucasian acute lymphoblastic leukaemia), caspase-3 is activated but independently of caspase-9. It appears that the effect of taxol on caspase-9 activation is also cell type-specific; for instance, taxolinduced apoptosis in the human leukemia HL-60 cell line has been observed to trigger the release of cytochrome c into the cytosol and induce Apaf-1-mediated caspase-3 and -9 activities [61, 62]. Park *et al.* (2004) and Lin *et al.* (2000) have demonstrated taxol-induced apoptosis through an ROS-independent pathway in human lymphoblastic leukemia cells and in hepatoma cells [63, 64].

Resistance to paclitaxel is defined as disease progression during treatment, failure to achieve tumour regression after a minimum of four courses of therapy, or recurrence of disease within 6 months of completion of paclitaxel therapy. The radio-sensitizing effects of docetaxel relative to paclitaxel have been evaluated in an *in vitro* comparative analysis using three human cancer cell lines (cervical cancer, mesothelioma, and lung cancer). Results showed that all three cell lines are more sensitive to docetaxel than to paclitaxel and that, although mesothelioma cells were intrinsically resistant to both radiation and taxanes, the resistance is partially overcome with administration of docetaxel before radiation [65-67]. Nevertheless, most patients with advanced lung cancer develop resistance to Taxol. In another study it was proven that paclitaxel, when combined with apoptin, can specifically inhibit expression resulting in additive cytotoxic activity in osteosarcoma and NSCLC cells [68]. A major impediment to paclitaxel cytotoxicity in NSCLC is the establishment of multi-drug resistance. In response to this, paclitaxel has been combined with other chemotherapeutic agents, resulting in increased response rates from 35 to > 50% [69, 70].

The involvement of caspases in taxane-induced cell death is cell line-dependent. Inhibition of caspase 1 and caspase 3 in a mouse cell line has been observed to prevent docetaxel-induced DNA fragmentation [71]. However, a pan caspase inhibitor failed to abolish decreases in cell viability by docetaxel in gastric cancer cells [72]. Moreover, taxanes have been found to cause apoptosis in caspase 3-deficient MCF-7 breast cancer cells [73]. Paclitaxel also activates caspase 8 in human colon cancer cells, suggesting a potential interaction between intrinsic and extrinsic apoptotic pathways [72].

### 2.3. Effects of Gefitinib

Gefitinib is an oral agent that is used in targeting the epidermal growth factor receptor tyrosine kinase, and it has a certain efficacy against non-small cell lung cancer [74]. EGFR-TKIs, gefitinib-IRESSA has been shown to inhibit cell proliferation which overexpresses EGFR, including some ER-negative cell lines [75, 76]. In Phase I trials, oral gefitinib tumour recession was observed in a variety of cancer types [77]. In Phase II studies, 500 mg/day of oral gefitinib used to treat breast cancer and 250 and 500 mg/day in NSCLC showed similar tolerability; however, in the case of NSCLC better tolerability was found in the lower dosage [78-80]. Gefitinib decreased tumour thyroid cancer cell growth *in vitro* and *in vivo* that overexpresses EGFR [81]. Since 2003, the FDA has approved three generations of EGFR tyrosine kinase inhibitor (TKI) including gefitinib, erlotinib, afatinib, and osimertinib [82, 83]. Gefitinib is a specific EGFR-TKI that is extensively applied to the treatment of non-small lung cells in clinical practice [84]. Gefitinib as a choice for first-line therapy significantly improved NSCLC patients' survival rate with EGFR mutation [85, 86]. Nonetheless, acquired resistance after gefitinib therapy is almost unavoidable and limits therapeutic success rate. To overcome resistance to EGFR-TKIs, a combination of them with other compounds has to be designed [87].

### 2.4. Effects of Gemcitabine

Gemcitabine *in vitro* and phase I studies has shown activity against many kinds of tumours, especially NSCLC [88-90]. However, this drug is highly cytotoxic and development of innate or acquired drug resistance has been a major challenge in gemcitabine therapy and has led to a reduction in the survival rate of cancer patients [91-93]. It is being used in combination with other drugs for the treatment of locally advanced or metastatic non-small-cell lung cancer, bladder cancer, and ovarian cancer [94]. Gemcitabine treatment showed greater inhibitory activity against breast cancer cells [95, 96]. In prostate cancer cells, a combination of gemcitabine with compounds αM and γM showed synergistic anti-cancer effects [97]. A combination of gemcitabine with other capecitabines such as cisplatin [98, 99], fluorouracil [100] and erlotinib[100], and more on stage III pancreatic cancer patients showed a slight synergistic effect. Therefore, development of the use of natural compounds to enhance gemcitabine in treating cancer is urgently needed.

### 2.5. Effects of Erlotinib

Erlotinib, like gefitinib, is a small molecular agent that is used to target epidermal growth factor receptors (EGFRs), and the inhibition of EGFR has become a main target for treating advanced non-small cell lung cancer (NSCLC) [101]. However, it is expensive and its efficacy is limited by primary or secondary drug resistance, which develops over extended periods of treatments [102]. Combined miR-34a and EGFR-TKIs synergistically sensitize both EGFR wild-type and mutant NSCLC cells [103].

## 3. Radiotherapy/chemoradiotherapy

Approximately one out of every three patients with NSCLC has a locally advanced tumor that is surgically unresectable [104]. Hence, radiotherapy remains a major therapeutic option for patients with such advanced lung cancer. Nevertheless, the effects of irradiation on malignant biological behaviours *(e.g.* migration and transformation of cancer cells) have yet to be fully clarified. However, we know that the median overall survival rate with radiotherapy alone is 9 to 11 months, and the 5-year survival rate is disappointingly low, at 3% to 10% [105-108]. Although radiotherapy is a major therapeutic modality for cancer treatment, previous findings have suggested that radiation promotes tumour migration, distant metastasis, and the invasive potential of cancer cells in disadvantage [109-111]. In contrast to SCLC cell lines, NSCLC cell lines are generally less sensitive to radiation and are poorly affected by current therapies, which means surgery represents almost the only curative therapy for about 25% of patients who are resectable at diagnosis [112].

On the other hand, pre-treatment with chemotherapy causes cancer cells to become sensitive to radiation therapy. Consequently, several studies have investigated the effects of combining radiotherapy with chemotherapy for patients with unresectable stage IIINSCLC [113]. Sause *et al.* (1995) have found similar results using a sequential approach, with a median overall survival rate of 13.8 months [114]. Zhang *et al.* (2010) have shown that wortmanin acts as a powerful radiosensitizer in NSCLC cells by inhibiting PI3K/Akt survival signalling and DNA-PKCs [115]. Similarly, gefitinib radiosensitizes NSCLC cells by inhibiting ataxi telangiectasia mutated (ATM) activity and thereby inducing mitotic cell death, and that COX-2 overexpression in NSCLC cells inhibits this action of gefitinib [116]. In addition, Schaake-Koning and colleagues administered cisplatin concurrently with radiotherapy to patients with nonmetastatic but inoperable NSCLC and demonstrated improved rates of survival and control of local disease as compared with radiotherapy alone [113]. However, this study reported increased toxicity, nausea, and vomiting in the concurrent chemoradiotherapy group. Therefore, it may be helpful in terms of lowering toxicity and enhancing the effect of radiation therapy if we can administer radiation therapy and natural compounds that can sensitize NSCLC for radiation therapy. Thus, searching for an effective sensitizer is becoming a hots topic and natural compounds including herbs and marine life are attractive and potentially viable alternatives to researchers.

## 4. Natural compounds

Natural compounds have long been a source of anticancer compounds. For many years, traditional Chinese medicines (TCM) have been applied for the treatment of cancers in China and beyond [117]. Herbal medicines are generally low in cost, plentiful, and show very little toxicity or side effects in clinical practice. Some of the most valuable compounds (such as paclitaxel and the Vinca alkaloids) were discovered either serendipitously or from slow and laborious *in vivo* screening [118]. Much of the current research in cancer therapeutics is aimed at developing drugs or vaccines to target key molecules that can inhibit tumour cell growth, metastasis, and proliferation. The cancer preventive and/ or protective activities of natural compounds lie in their effects on cellular defences like detoxifying and antioxidant enzyme systems, and the induction of anti-inflammatory and antitumor or anti-metastasis responses, often by targeting specific key transcription factors [119].

In clinical treatment, most NSCLC patients respond poorly to conventional chemotherapy because of the emergence of resistance. Hence, there is an urgent need to develop novel treatment strategies to improve the sensitivity of cancer cells to chemotherapy-induced cell death. Below we present some examples of how apoptosis pathways are targeted by select naturally occurring agents and how these events can be exploited for cancer therapy.

### 4.1. Wortmannin and Roscovitine

The purine analogues roscovitine is a small molecule that inhibits the activity of cyclin-dependent kinases (CDKs) *via* direct competition in the ATP-binding site. It is particularly active against Cdk1 (Cdc2), Cdk2, and Cdk5 and induces G1 and G2-M cell cycle arrest [120]. Roscovitine has been reported to have antitumour effects in different cancer cell lines [121-123]. Similarly, roscovitine induces apoptosis in A549 cells in a dose-dependent manner. Meanwhile, wortmannin, a fungal metabolite, is a potent specific PI3K inhibitor, which binds to the p110 catalytic subunit of PI3K and irreversibly inhibits the enzyme [124], something which could chemosensitize three human tumour cell lines (A549, HCT116 and HeLa cells). In A549 cells, wortmannin increases roscovitine-induced apoptosis in a dose-dependent manner, which is correlated with the inhibition of phosphorylated PKB/Akt level. Wortmannin enhances the effects of roscovitine by causing a pronounced reduction of mitochondrial membrane potential (MMP) and increases of cytochrome c release and active caspase-3, as well as enhances the activation of Bax and Bad, including Bax oligomerization and the mitochondrial translocation of Bax and Bad. Taken together, these results provide evidence for the potential application of a roscovitine and wormannin combination in clinical treatment for solid tumours [125].

### 4.2. Cordyceps militaris

*Cordyceps militaris* is well known as a traditional medicinal mushroom and is a potentially interesting candidate for use in cancer treatment. Water extract of *C. militaris* (WECM) induces the apoptosis of A549 cells through a signalling cascade of death receptor-mediated extrinsic and mitochondria-mediated intrinsic caspase pathways. It has also been concluded that apoptotic events due to WECM are mediated with diminished telomerase activity through the inhibition of hTERT transcriptional activity [126].

### 4.3. Resveratrol

Resveratrol has been assessed in over 110 clinical trials, such as in DM and metabolic syndrome patients and certain types of cancer patients [127]. Resveratrol (3,5,4'-trihydroxy-stilbene) is a phytoalexin found in red wine and a variety of plants, including grapes, peanuts, mulberries, and legumes and are produced due to stress, injury, fungal infection, or UV exposure [128, 129]. Resveratrol induces antioxidant and anti-inflammatory effects and also has been found to inhibit the proliferation of a various cancer cells [130, 131]. What’s more, resveratrol has been found to inhibit platelet aggregation [132] and also to have antioxidant properties [133]. Resveratrol is reported to have protective effects against lung cancer; it alters a large number of genes and proteins and inhibits A549 cell proliferation by inducing cell cycle arrest, inducing apoptosis, and by altering the intracellular Smad signalling of the TGF-β pathway [134]. Resveratrol has already been established as an antiproliferative agent in A549 human lung cancer cells, and this effect has been correlated with the suppression of the phosphorylation of Rb protein and transcription factors such as nuclear factor-kB (NF-kB) and activator protein-1 [135]. It has been identified that resveratrol administration in colorectal adenocarcinoma patients reduces tumour cell proliferation [136].

### 4.4. OSU03013

OSU03013 is a derivative of celecoxib. Although celecoxib is an inhibitor of cyclooxygenase (COX)-2, substantial data indicate that celecoxib-induced apoptosis cell death occurs through a COX-2–independent pathway [137]. A recent study indicated that OSU03013 can induce apoptosis in prostate cancer cells through the 3-phosphoinositide–dependent kinase 1 (PDK1)/AKT signalling pathway and may more strongly inhibit cell growth than celecoxib [138]. In addition, OSU03013 has been used in breast cancer treatment, and it has been found to have a higher cytotoxicity especially in breast cancer cells with epidermal growth factor receptor (HER)-2 overexpression [139]. Tong *et al.* (2006) have found that 10 μM of OSU03013 can induce cytochrome C-mediated apoptosis in A549 lung cancer cells especially at low concentrations of exogenous-expressed AKT [140]. Similarly, Tan *et al.* (2008) found that OSU03013 can affect several pathways such as the cAMP-dependent protein kinase (PKA) and Wnt/h catenin pathways and cause ER stress–induced apoptosis at a dose as low as 2 μM in lung cancer cells [141].

### 4.5. Myricetin

Myricetin, a flavonoid commonly found in tea, wines, berries, fruits, and medicinal plants, has been reported to possess antioxidative, antiproliferative, and anti-inflammatory qualities. Previous studies have shown that myricetin exerts an antiproliferative effect on lung, esophageal, leukemia, and prostate cancer cells [142-144]. Myricetin may act as a direct antioxidant that scavenges or quenches oxygen free radicals, and as an indirect antioxidant that induces antioxidant enzymes to protect cells against H_2_O_2_-induced cell damage [145].

### 4.6. Berberine

Berberine is an isoquinoline derivative alkaloid isolated from many medicinal herbs, such as *Hydrastis canadensis, Cortex phellodendri,* and *Rhizoma coptidis.* It is widely used in Traditional Chinese medicine for the treatment of inflammatory diseases and anti-microbial activities [146-149]. Berberine has been reported to have a wide range of pharmacological effects, including interaction with DNA to form complexes, inhibition of DNA and protein synthesis, an arresting effect on cell cycle progress, an inhibition of tumour cell proliferation, and an anticancer effect. It has been reported that berberine decreases the motility and invasion of non-small lung cancer cells by alleviating the activation of c-Fos, c-Jun, and NF-κB, and thus inhibits uPA, MMP2 proteins [150]. Berberine exerts an antitumor effect *via* inhibition of cell proliferation and induction of apoptosis in ovarian cancer cells[151]. Cotreatment of Curcumin and Berberine has displayed synergistic chemopreventive effects *via* inducing caspase-dependent apoptosis and autophagic cell death through ERK and JNK/Beclin1/Bcl-2 signalling pathways, respectively, in breast cancer cell lines [152].

### 4.7. Antroquinonol

Antroquinonol, a ubiquinone derivative isolated from mycelia and the fruiting bodies of *A. camphorata* has been reported to exhibit cytotoxic activities against cancer cell lines MCF-7, MDA-MB-231, Hep 3B, Hep G2 and DU-145, LNCaP with the IC_50_ values ranging from 0.13 to 6.09 μM [153]. Along with other groups, we have have found that antroquinonol inhibits lung cancer and liver cancer cells by modulating the AMP-activated protein kinase (AMPK) or phosphatidylinositol-3-kinase (PI3K)/ mammalian target of rapamycin (mTOR) pathways [154, 155]. Recent studies have reported that Antroquinonol induces apoptosis and autophagy of pancreatic cancer cells [156]. In colon cancer, ANQ has been found to suppress stem cell-like properties by targeting PI3K/AKT/β-catenin signalling [157].

## 5. Conclusion

Surgery or radiotherapy is the standard option for patients with early stages of NSCLC. Chemotherapy has shown some benefit when used alone in patients with stage IV of the disease, as well as in combination with radiotherapy in patients with locally advanced disease and in the preoperative setting in those with early stages of NSCLC. Platinum drugs are still considered of crucial interest based on clinical studies and the results of meta-analyses, with their inconvenience being their observed toxicity and the inherent resistance. The poor efficacy and considerable toxicity of chemotherapy has caused great pessimism for many years regarding this approach, as only a small positive impact on survival rates was observed. Chemotherapy is now a broadly accepted form of therapy for stage IIIB/IV NSCLC, and there is growing interest in its use in earlier stages of the disease when combined with other (local) therapy. Meanwhile, natural compounds have been used to treat various diseases and are becoming a significant research area for drug discovery. Using natural agents along with chemotherapy drugs in patients with advanced and/or refractory solid tumours could reduce the toxicity risk produced by chemotherapy, and this could be an accessible approach to cancer control and management.

## Conflicts of Interest

The authors wish to declare they have no conflicts of interest.
